# The Egg Yolk Content in ω-3 and Conjugated Fatty Acids Can Be Sustainably Increased upon Long-Term Feeding of Laying Hens with a Diet Containing Flaxseeds and Pomegranate Seed Oil

**DOI:** 10.3390/foods10051134

**Published:** 2021-05-19

**Authors:** Monique T. Ngo Njembe, Louis Dejonghe, Eleonore Verstraelen, Eric Mignolet, Matthieu Leclercq, Hélène Dailly, Cécile Gardin, Marine Buchet, Caroline Waingeh Nain, Yvan Larondelle

**Affiliations:** 1 Louvain Institute of Biomolecular Science and Technology, UCLouvain, 1348 Louvain-la-Neuve, Belgium; monique.ngonjembe@uclouvain.be (M.T.N.N.); louis.dejonghe@uclouvain.be (L.D.); eleonore.verstraelen@uclouvain.be (E.V.); eric.mignolet@uclouvain.be (E.M.); cecile.gardin@uclouvain.be (C.G.); marine.buchet@uclouvain.be (M.B.); caroline.nain@uclouvain.be (C.W.N.); 2 Earth and Life Institute, MOCA Platform, UCLouvain, 1348 Louvain-la-Neuve, Belgium; matthieu.leclercq@uclouvain.be (M.L.); helene.dailly@uclouvain.be (H.D.)

**Keywords:** enriched eggs, omega-3 fatty acids, conjugated fatty acids, flaxseed, pomegranate seed oil, alpha-linolenic acid, docosahexaenoic acid, punicic acid, rumenic acid

## Abstract

Long-term feeding trials examining the incorporation of conjugated linolenic acids (CLnA) into the diet of laying hens are lacking. In the present study, we compared two diets in sixty-six red Sex-Link hens (33 hens/treatment), fed for 26 weeks. The control diet was high in oleic acid, while the test diet was high in α-linolenic acid (ALA) and punicic acid (PunA). No significant differences were observed between treatments for hens’ performance, egg weight and yolk weight. In contrast, dietary ALA and PunA resulted in a significant increase in n-3 PUFA, rumenic acid (RmA) and PunA contents in egg yolk, as well as in the liver, heart, muscle and adipose tissue of the hens. Other conjugated dienes resulting from the metabolism of PunA or RmA also accumulated in the egg yolk and tissues. Unlike DHA, which was exclusively distributed in phospholipids, ALA, RmA and PunA were preferably distributed in triglycerides.

## 1. Introduction

One of the fascinating findings in human nutrition is that certain foods, beyond providing essential nutrients to the body, contain high concentrations of biologically active compounds that are effective in preventing or treating chronic or non-communicable diseases, among which cardiovascular diseases, diabetes and cancers [1,2]. As a result, nutritional strategies have been extensively developed to enrich widely consumed foods with such beneficial compounds, in order to increase their dietary intake without drastic changes in diet or eating habits. In this sense, chicken eggs have been found as an excellent candidate for nutritional enrichment.

As one of the cheapest sources of animal protein and fat consumed all over the world, eggs are a significant component of the human diet. Moreover, by modifying the feed of laying hens it is possible to significantly increase the levels of certain nutrients in the egg to such an extent that consuming a single egg could provide these nutrients in relevant amounts, as compared to the recommended daily allowance [3]. For example, plant seeds rich in α-linolenic acid (ALA, C18:3c9,c12,c15), such as flaxseeds, have been commonly added to hens’ diet to enrich eggs mainly with ALA and also, to some extent, with one of its long-chain anabolic derivative, docosahexaenoic acid (DHA, C22:6c4,c7,c10,c13,c16,c19). Omega-3 polyunsaturated fatty acids (n-3 PUFA) have shown health benefits in terms of inflammatory processes [4,5] and neurodegenerative diseases [6]. Long-chain n-3 PUFA but not ALA have been associated with a low risk of cardiovascular diseases [7,8]. In addition, DHA is known to be of utmost importance for the proper development and functioning of the nervous system [9]. Conjugated linoleic acids (CLA) and conjugated linolenic acids (CLnA) have been shown to have a wide range of biological activities, among which anti-diabetic [10,11], anti-inflammatory [12] and anti-cancer properties [13,14]. The bulk of CLA is supplied to humans by the diet, especially through the consumption of dairy products and ruminant meat [15], but dietary intake remains relatively low as CLA account for less than 2% of total ruminant fat. On the other hand, CLnA are present in large amounts in the oil of certain plant seeds, such as pomegranate, ricinodendron, bitter gourd or catalpa [16]. A Δ13-reductase activity allowing the conversion of certain CLnA into CLA has been found in animal and human tissues [17,18,19]. We have previously shown that dietary sources of CLnA such as pomegranate seed oil (PSO), high in punicic acid (PunA, C18:3c9,t11,c13), supplemented in the hen’s diet allowed a considerable increase in the egg content of PunA, as well as rumenic acid (RmA, C18:2c9,t11), an omega-7 CLA [20]. Thus, feeding laying hens with diets containing CLnA is a means for incorporating both CLA and CLnA into the egg yolk fat.

Yet, there have been few studies on CLnA dietary supplementation in laying hens, and those reported were short-term feeding trials (less than 3-month long). In addition, they did not address the fate of CLnA in hens’ tissues. However, studies in rodents have indicated that CLnA are incorporated in animal tissues as CLA, and the predominant form is RmA [21,22]. Besides, this accumulation of RmA goes along with a decrease in total omega-6 (n-6) PUFA in heart and adipose tissue and an increase in total n-3 PUFA in the liver and heart [23]. This suggests that feeding the hens with PunA might also alter the fatty acid profile of their tissues. Therefore, understanding the effects of a CLnA-rich diet on the fatty acid composition of hens’ tissues is an important consideration in designing an effective enrichment program.

In a previous study, we reported the effect of the dietary combination of flaxseeds and PSO on the fatty acid profile of eggs during a short period of the laying cycle (12 to 21 days) using a reduced number of individually housed hens (4 hens/diet) [20]. For the current study, the objective was to determine the performance of the hens, and the incorporation of fatty acids in the egg yolk over a fairly long production period (26 weeks) under regular conditions of an egg production unit, with a larger number of hens housed in the same pen. In the current assessment, we examine the interaction between the length of the feeding period and the levels of ALA, DHA, RmA and PunA in eggs, as well as the distribution of these fatty acids into lipid classes. We also report on the presence of conjugated dienes resulting from the metabolism of PunA and/or RmA in egg yolk. In addition, we determine how a 26-week long supplementation with dietary flaxseeds and PSO affects the fatty acid composition of different hens’ tissues.

## 2. Materials and Methods

### 2.1. Ethics Statement

The data presented in this paper are associated with the study entitled “A three-month consumption of eggs enriched with ω-3, ω-5 and ω-7 polyunsaturated fatty acids significantly decreases the waist circumference of subjects at risk of developing metabolic syndrome: a double-blind randomized controlled trial” [24]. The animals used in the framework of this study were raised in compliance with animal care and welfare directives at the University Farm of UCLouvain (Corroy-le-Grand, Belgium).

### 2.2. Birds, Housing and Diets

Sixty-six 5-month-old red Sex-Link hens obtained from the Limal poultry farm (Wavre, Belgium) were housed in floor pens maintained under controlled temperature and lighting conditions as described previously [20], and fed with a standard feed for egg-producing hens (Aveve, Leuven, Belgium). At 7 months of age, the hens were randomly divided into two separate lots, and assigned to two different diets (33 hens/diet). One group was fed the standard feed supplemented with 10 wt % extra virgin olive oil obtained from a local commercial source (F-OL diet). The other group received the standard feed added with 7.8 wt % flaxseeds (Dumoulin, Kortrijk, Belgium) and 7 wt %. PSO (Ol’vita, Pszenno, Poland) (FS-PSO diet). The diets were formulated to differ in fatty acid composition and contained a high amount of either oleic acid (OA, C18:1c9) (F-OL diet) or ALA and PunA (FS-PSO diet). The inclusion rates for olive oil and the mix of flaxseeds and PSO were made up to equalize the total fat content of each diet at 15%. Fresh diets were prepared daily to minimize oxidation of the fats. Feed samples from each diet were taken after mixing, and analyzed as described later. The fatty acid composition of the diets is shown in Table 1. The dietary treatments were administered for 26 weeks, and all animals had free access to food and water.

### 2.3. Sample Collection and Measurements

Eggs were harvested daily throughout the study from fourteen days after the start of the experimental feeding to allow substantial deposition of fatty acids in the egg yolk. Egg production was calculated from the total of eggs divided by the number of days and hens, and expressed as average hen’s production per day. The fatty acid composition of the eggs was determined at weekly interval until the end of the trial from six eggs randomly selected in each treatment. The eggs were weighed whole and then cracked. The yolks were separated from the albumen and the shell, and weighed individually. Results of four consecutive weeks were grouped to build up 6 periods of 28 days each. At the end of the feeding period, five birds from each treatment group were randomly selected and euthanized by exsanguination. Liver, heart, adipose tissue and abdominal muscles were collected and kept frozen at −20 °C for further fatty acid analysis.

### 2.4. Lipid Extraction and Fatty Acid Analysis

The fatty acid composition of feeds, hens’ tissues and egg yolks was evaluated after chloroform-methanol-water extraction of the lipids from the samples. The Folch method [25] was used for feed and egg yolk samples, and tissue lipids were extracted by the Bligh and Dyer method [26]. Lipids extracted from tissues and some yolk samples were separated by solid phase extraction (SPE) into three lipid fractions: triglycerides (TG), free fatty acids (FFA) and phospholipids (PL), using a Bond Elut-NH2, 200 mg, 3 mL silica-based SPE column (Agilent technologies, Santa Clara, CA, USA), as described previously [17]. Fatty acids (extracted from total lipids and lipid fractions) were converted into fatty acid methyl esters (FAME) via methylation under alkaline conditions (KOH/MeOH, 0.1 M, at 70 °C for 60 min) and then under acidic conditions (HCl/MeOH, 1.2 M, at 70 °C for 20 min). The resultant FAME were subsequently extracted using hexane (VWR Chemicals, Radnor, PA, USA) and separated by gas chromatography (GC).

### 2.5. Identification and Quantification of Fatty Acids by GC–FID and –MS

GC analysis was performed using a Trace 1310 GC (ThermoFisher scientific, Waltham, MA, USA) equipped with an RT2560 capillary column (100 m × 0.25 mm, 0.2 µm film thickness) (Restek, Lisses, France), an automatic injector and a flame ionization detector (FID) set at 255 °C. Samples were injected with hydrogen as carrier gas at a constant pressure of 200 kPa into the column programed for ramped oven temperatures as described previously [20]. FAME were identified by comparing their retention times to authentic standards of known composition (Larodan, Solna, Sweden). Peak areas and fatty acid concentrations were calculated using the ChromQuest 5.0 software (ThermoFisher Scientific).

The lipid extracts were also analyzed by GC-MS to characterize fatty acid peaks that could not be identified with GC-FID and to confirm the identification of the fatty acids detected. For GC-MS analysis, a Trace 1310 GC was equipped with an ISQ QD mass spectrometer (MS) detector (ThermoFisher Scientific, Waltham, MA, USA). The column and the oven temperature program were identical to the GC-FID analysis. The column operating conditions of the GC-MS analysis were as follows: helium carrier gas at 2 mL/min; a split/splitless inlet at 250 °C; a split ratio of 10:1; a transfer line temperature of 250 °C. The parameters used for mass spectrometry analyses were as follows: ionization energy at 70 eV; ion source temperature of 300 °C; dwell time of 0.2 s; a mass range of 40–400 m/z. The system operated and the data were processed using the Thermo Scientific Chromeleon 7 Chromatography Data System (CDS) software. Fatty acid values were expressed as milligrams per gram of material (mg/g) or as a percentage (%) of the total fatty acids identified.

### 2.6. Statistical Analysis

Egg weight, yolk weight and fatty acid composition of egg yolks were analyzed by two-way analysis of variance (ANOVA) using JMP 15 (SAS institute inc., Cary, NC, USA), to determine the effects of dietary treatments, the duration of the experiment and their interaction. Treatments (F-OL and FS-PSO) and periods (1 to 6) were the fixed effects and least squares means, adjusted using the Tukey–Kramer test, were compared for significant differences set at *p* < 0.05. The difference between dietary treatments in terms of fatty acids in tissues was determined using a two-tailed *t*-test and the results were expressed as means and standard errors of the mean.

## 3. Results

### 3.1. Dietary Fats and Hens’ Performance

CLnA were only present in the PSO-supplemented diet (Table 1). The predominantly abundant CLnA was PunA, accounting for approximately 73% of the total fatty acids in PSO. Its concentration was 39.53 mg/g in the FS-PSO diet. Five other CLnA present at low levels in PSO, α-eleostearic acid (C18:3c9,t11,t13), α-calendic acid (C18:3t8,t10,c12), catalpic acid (C18:3t9,t11,c13), β-eleostearic acid (C18:3t9,t11,t13), and β-calendic acid (C18:3t8,t10,t12), were detected in the hens’ diet. Inclusion of flaxseeds resulted in the incorporation of a high level of ALA in the FS-PSO diet. The F-OL diet had a very high content of OA (91.06 mg/g). Other fatty acids such as palmitic acid (C16:0), stearic acid (C18:0), and linoleic acid (C18:2c9,c12) were also higher in the F-OL diet.

Egg production was similar in hens from the two dietary treatments, averaging about 85%, over the entire experimental period (Table 2). Similarly, egg weight and yolk weight were not affected by the dietary treatments or the duration of feeding. Total fat was also similar in yolks from the two dietary treatments (as mean ± SEM: F-OL = 31.08 ± 0.59%; FS-PSO = 32.45 ± 0.44%).

### 3.2. Fatty Acid Composition of Egg Yolks

The fatty acid compositions of the yolks (in mg/g of yolk) as a function of the two dietary treatments and over the entire feeding duration are presented in Table 3. Total saturated fatty acids (SFA) showed a highly significant (*p* ˂ 0.0001) time effect for both F-OL and FS-PSO diets. Palmitic and stearic acids decreased in the yolks in both groups all along the experiment, with the lowest levels seen at period 6. However, higher concentrations of these fatty acids were observed with the FS-PSO diet compared to F-OL.

As compared with the FS-PSO group, feeding the F-OL diet to hens resulted in higher concentrations of hypogeic acid (C16:1c7), OA and cis-vaccenic acid (C18:1c11) in yolk lipids. Unlike other monounsaturated fatty acids (MUFA), there was no significant effect of the diet on palmitoleic acid (C16:1c9), whose levels within periods were not significantly different between treatment groups throughout the experiment. The duration of the experiment resulted in a further decrease in MUFA of FS-PSO egg yolks. The same observation was made in F-OL yolks for palmitoleic and cis-vaccenic acids. In contrast, there were no significant differences in OA levels in F-OL eggs throughout the feeding duration.

There was an effect of treatment on the amount of total n-6 PUFA in egg yolks, although there was no interaction between dietary treatment and duration of feeding. However, the treatment effect varied depending on the n-6 fatty acids. The accumulation of linoleic acid (C18:2c9,c12) was reduced in both egg types. The decrease was greater in F-OL eggs while the diet contained a higher amount of this fatty acid (Table 1). The accumulations of arachidonic acid (C20:4c5,c8,c11,c14) and n-6 docosapentaenoic acid (n-6 DPA; C22:5c4,c7,c10,c13,c16) were lower in FS-PSO eggs, whereas their concentrations remained relatively constant in F-OL eggs throughout the experiment.

The concentration of ALA increased in yolks of hens fed on flaxseeds and this increase persisted until the end of the experiment. In contrast, ALA in the yolks produced by hens fed the F-OL diet remained at a relatively low concentration throughout the experiment and tended to decrease. Including olive oil in the diet did not affect DHA concentration in yolks of the F-OL group, and the accumulation of synthesized DHA from ALA was 2.2-fold higher in the yolk of FS-PSO eggs compared to F-OL. The effects of dietary treatments on n-3 DPA (C22:5c7,c10,c13,c16,c19) concentrations in yolk lipids were similar to those seen with DHA, namely an increase in FS-PSO eggs and no significant change in F-OL eggs. A significant (*p* < 0.0001) diet effect was observed in the deposition of eicosapentaenoic acid (EPA, C20:5c5,c8,c11,c14,c17), although this fatty acid was absent in F-OL eggs and deposited in very low concentrations in FS-PSO yolk lipids. There was no time effect on the total concentration of n-3 PUFA in egg yolks.

Levels of CLnA in egg lipids were greatly (*p* < 0.0001) influenced by the incorporation of PSO in the diet, with PunA, found only in eggs from the FS-PSO group. CLnA other than PunA were also deposited in yolk lipids. Expressed as PunA equivalents, the concentrations of these CLnA, namely α-eleostearic acid (C18:3c9,t11,t13), α-calendic acid (C18:3t8,t10,c12), catalpic acid (C18:3t9,t11,c13), β-eleostearic acid (C18:3t9,t11,t13), and β-calendic acid (C18:3t8,t10,t12) clearly reflected their levels in the FS-PSO diet. Another CLnA, cis-6, cis-9, trans-11-octadecatrienoic acid (c6,c9,t11-CLnA (C18:3c6,c9,t11)), not identified in the FS-PSO diet, was detected in egg yolk lipids. Its level significantly increased in the FS-PSO group as compared to trace amounts in the F-OL group. Regarding the CLA isomers, the concentration of RmA in the egg yolks of the FS-PSO group was drastically higher than in the F-OL group. Furthermore, it increased as the experiment progressed. The maximum concentration (40.19 mg/g of yolk) was seen at period 6. Some other conjugated dienoic acids, including cis-7, trans-9-hexadecadienoic acid (C16:2c7,t9) and trans-9, trans-11-octadecadienoic acid (t9,t11-CLA (C18:2t9,t11)) were detected in egg yolks. While remaining considerably lower compared to RmA, their concentrations were largely higher in FS-PSO eggs than in F-OL eggs where they remained in trace amounts.

### 3.3. Distribution of OA, ALA, RmA and PunA of Yolk into Lipid Classes

**Figure 1:**
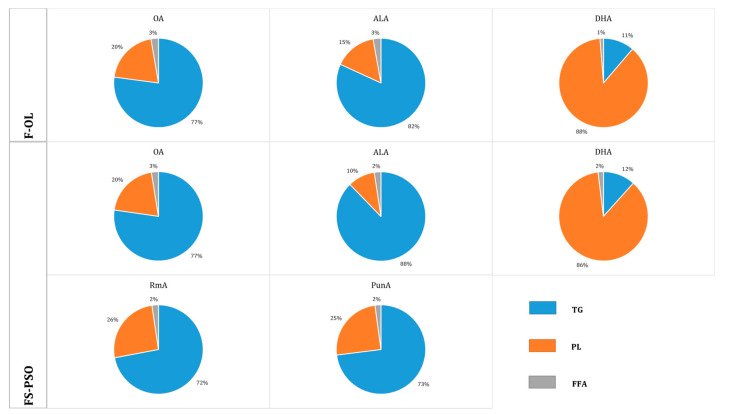
Incorporation of oleic acid (OA), α-linolenic acid (ALA), docosahexaenoic acid (DHA), rumenic acid (RmA) and punicic acid (PunA) into different lipid fractions (triglycerides (TG), phospholipids (PL), and free fatty acids (FFA)) of egg yolk. F-OL: hens’ feed supplemented with 10 wt % olive oil. FS-PSO: hens’ feed supplemented with 7.8 wt % flaxseeds and 7 wt % pomegranate seed oil.

Figure 1 shows the distribution of OA, ALA and DHA, as well as that of RmA and PunA in the case of FS-PSO eggs, into the different lipid classes (TG, PL and FFA), and Table 4 presents the fatty acid composition of TG and PL in percentages by weight of total fatty acids. Fatty acids in yolk were deposited primarily in TG and PL, which accounted for 60% to 65% and 29% to 33% of yolk lipids, respectively. The distribution of OA was the same in lipid classes (TG, 77%; PL, 20%; FFA, 3%) for both types of eggs. However, OA proportions in TG and PL in F-OL eggs were two times higher than in FS-PSO eggs. The enrichment of ALA upon feeding flaxseeds was mainly in yolk TG, 88% of ALA being deposited in the TG fraction, resulting in an average proportion of 2.89% ALA in the total fatty acids in TG. Only 10% of ALA was accumulated in the PL fraction of FS-PSO yolks. The low ALA content in F-OL eggs exhibited almost the same distribution pattern (TG, 82% and PL, 15%). In contrast, 87% of the DHA accumulated was found in the PL fraction regardless of its concentration in the yolk. Supplementation of the hens’ diet with PSO resulted in the accumulation of PunA and RmA mainly in the TG fraction of yolk lipids. Besides, these two fatty acids were distributed in similar pattern between the different lipid classes. Indeed, 73% of PunA and 72% of RmA were accumulated in the TG fraction, whereas only 25% of PunA and 26% of RmA were found in the PL fraction.

### 3.4. Incorporation of Dietary Lipids on Tissues of Hens

Analysis of the liver, heart, muscle and adipose tissue of the hens showed that the composition of fatty acids in these tissues was greatly affected by dietary lipids (Table 5). In general, palmitic acid, OA and linoleic acid were the major components of both TG and PL fractions. Stearic acid and long chain PUFA were present in significant amounts in PL, but only very small amounts of these fatty acids were detectable in TG. Unlike ALA, which accumulated almost exclusively in TG, RmA and PunA were deposited in appreciable proportions in both TG and PL fractions.

As observed with eggs, the CLnA present in the feed were incorporated into the hens’ tissues from the FS-PSO group in proportions corresponding to those provided by the diet. Apart from PunA, the other food-borne CLnA were at trace levels. PunA, together with c6,c9,t11-CLnA, were the isomers found in substantial amounts in hens’ tissues. In addition, the proportions of c6,c9,t11-CLnA were higher than that of PunA in the PL fraction in tissues. CLA were also present in all the tissues examined. RmA was the predominant CLA isomer and it was found in tissue fat at higher levels than PunA. In contrast, no CLnA was detected in the tissues of animals fed with the F-OL diet, whereas CLA were detected at very low levels.

There was a substantial increase in OA in all tissues in the group receiving the F-OL diet. Palmitoleic acid was similar between both dietary treatments, while hypogeic and cis-vaccenic acids were higher in hens fed the F-OL diet. Among SFA, there was no difference in the amounts of palmitic and stearic acids in muscle. In contrast, the proportions of these two fatty acids were significantly increased in the heart and adipose tissue of birds fed with the FS-PSO diet. In the liver, the contents of palmitic and stearic acids were also increased in the FS-PSO group, although the increase in palmitic acid was not significant. ALA was higher in the fat of hens fed flaxseeds, resulting in a significant increase in the total n-3 PUFA proportion in the TG fraction. In parallel, a significant increase in the long-chain n-3 PUFA, DHA and n-3 DPA, was seen in the PL fraction, when compared with the F-OL group. EPA was found at very low levels in both groups. Linoleic acid significantly accumulated in the fat of all tissues when feeding the FS-PSO diet. In contrast, the proportions of the long-chain n-6 PUFA, arachidonic acid and n-6 DPA, were significantly reduced in the PL fraction compared to the F-OL group.

## 4. Discussion

The inclusion in hens’ feed of flaxseeds, a common ingredient for the enrichment of eggs in n-3 PUFA, together with an oil rich in CLnA, as a precursor for the endogenous formation of CLA, is a dietary feeding strategy to advantageously modify the fatty acid composition of eggs. We assessed the enrichment by comparison with olive oil, which was incorporated in a diet low in ALA.

Egg production, as well as egg and yolk weights were unaffected by either dietary treatment or duration of feeding. However, egg weight, as well as yolk weight tended to increase with hens’ age, as expected [27]. Consistent with our results, Bean and Leeson [28] reported no significant effect of feeding a flaxseed-based diet on egg production, egg weight and yolk weight between 28 and 53 weeks of age. Kostogrys et al. [29] indicated that using PSO to naturally enrich eggs with CLA and CLnA did not alter egg quality parameters, as well as organoleptic properties. In this study, the physico-chemical and sensory properties of the eggs were not analyzed. However, eggs have been tested for regular consumption for 3 months by a panel of egg eaters. No adverse effects of enrichment on egg quality were reported [24].

The results obtained on the enrichment of eggs in n-3 PUFA, RmA and PunA are in agreement with our previous reported results and others [20,29]. The amount of ALA and DHA deposition in eggs through the inclusion of flaxseeds in the FS-PSO diet was significantly improved, while a lower level of ALA intake supplied by the F-OL diet led to the accumulation of much less ALA and DHA in eggs, with no variation with the duration of feeding. We have previously shown that changing the fatty acid profile of eggs in response to a dietary modification is a gradual process. When the hens were monitored individually, the steady-state deposition of RmA and a stable fatty acid profile in the egg yolk were achieved within two weeks of feeding, for the laying hen to adapt to the enriched diet and reach a plateau of transfer of dietary fatty acids into developing ovarian follicles [20]. In the present study, the inclusion of PSO resulted in a substantial accumulation of PunA and RmA in FS-PSO eggs. Interestingly, the stability of RmA enrichment in FS-PSO eggs was not achieved within one month of feeding. In addition, the upward trend observed over the feeding period implies that the maximal accumulation level of RmA in eggs might not have been totally reached, even after 26 weeks.

The incorporation of PSO in the feed of the hens also allowed the deposition to some extent of another CLA isomer (t9,t11-CLA), which probably derived from the metabolization of CLnA other than PunA contained in PSO. Tsuzuki et al. [19,30] reported that PunA and α-eleostearic acid are converted in RmA by a Δ13 saturation reaction carried out by a nicotinamide adenine dinucleotide phosphate (NADPH)-dependent enzyme, which is a novel enzyme recognizing conjugated trienoic acids or the enzyme active in the leukotriene B4 reductive pathway. Suzuki et al. [31] detected t9,t11-CLA in the liver tissue and colonic mucosa of rats fed a diet containing *Catalpa ovata* seed oil, an oil rich in catalpic acid. Additionally, Schneider et al. [17] showed that the CLA isomer produced from β-eleostearic acid and catalpic acid is t9,t11-CLA. These results indicate that the hen is able to efficiently convert various CLnA isomers with a Δ13 double bond.

Feeding PSO along with flaxseeds to hens increased the concentration of linoleic acid in FS-PSO eggs but decrease the level of arachidonic acid. The competition between n-6 PUFA and n-3 PUFA substrates, linoleic acid and ALA, respectively, to use the same enzymatic machinery for the bioconversion into longer chain PUFA is well known. The conversion of linoleic acid to arachidonic acid and ALA to EPA requires an alternating sequence of chain desaturation and elongation reactions catalyzed by ∆6- and ∆5-desaturases, and elongases. ALA is preferably metabolized over linoleic acid by these enzymes [32]. In birds consuming ALA, long-chain n-6-PUFA (arachidonic acid and n-6 DPA) decreased and were replaced by long-chain n-3 PUFA (EPA, n-3 DPA and DHA). On the other hand, RmA has been shown to undergo elongation and desaturation processes similar to those that occur with linoleic acid, maintaining the conjugated diene structure. Thus, RmA underwent a ∆6-desaturation leading to the formation of c6,c9,t11-CLnA. We failed to detect other long-chain conjugated diene derivatives. Likely, their levels were too low to accumulate in the yolk. Furthermore, cis-7, trans-9-hexadecadienoic acid found in FS-PSO eggs is probably a conjugated C16:2 metabolite derived from peroxisomal β-oxidation of RmA [33].

Concerning SFA and MUFA, the current results are consistent with other studies indicating that PunA and RmA have an inhibitory action on Δ9-desaturase [20,29,34], leading to an increase in SFA. Although feeding with the F-OL diet significantly increased OA in eggs produced by hens in this group as compared to the FS-PSO group, the negative effect of PunA and RmA on MUFA accumulation could be observed through a significant decline in FS-PSO eggs over the course of the feeding period.

Similar to other monogastric animals, laying hens have a limited endogenous enzymatic ability to modify the structure of dietary fatty acids [35]. The level of fatty acids in the yolk is finite due to the total fat content of 30% to 33% and reaching a plateau of saturation or a stable state of accumulation for a fatty acid in the yolk is directly influenced by the nature and the concentration of dietary fatty acids. The current results showed that the stability of the fatty acid composition of the egg yolk for either the F-OL group or the FS-PSO group was achievable after one month of feeding. During post-absorptive metabolism, fatty acids are deposited in the egg yolk as TG for long-term energy storage, or as PL mobilized more rapidly during embryonic tissue development [36]. The different dietary supplementations had no effect on the distribution of OA, ALA and DHA in the various lipid fractions. Moreover, PunA and RmA were mostly deposited in the TG fraction according to distribution patterns that were closer to that of OA than to ALA.

The fatty acid profiles of hens’ tissues reflected those of the diets with significant levels of OA in the F-OL group, and ALA and PunA in the FS-PSO group. Similar to eggs, an accumulation of DHA and RmA, an increase in SFA and a decrease in MUFA related to the metabolization of ALA and PunA were observed in tissue lipids of the FS-PSO group. This suggests that the entire lipid metabolism in the hen was altered. As could be expected, the fatty acid composition of the TG and PL lipid classes in hens’ tissues was similar to that of egg yolk. PunA and RmA in the hens’ tissues were preferentially stored as an energy source in the TG fraction. This distribution pattern is also true for other food-borne CLnA, depending on their supply through the diet. Unlike its non-conjugated counterpart, ALA, PunA was also deposited in appreciable amount in the PL fraction. Likewise, RmA and even more its metabolic derivative c6,c9,t11-CLnA were not only found at high levels in the TG fraction of tissue lipids, but were also incorporated into cell membrane phospholipids. This result is consistent with those of Banni and co-investigators, reporting in rats that RmA and its desaturation metabolite, the conjugated diene C18:3, are incorporated into liver phospholipids in a proportion ranging from 18% to 31% of their total amount. They also found that these two fatty acids were predominantly present in the phosphatidylcholine fraction [37]. This last observation cannot be confirmed in our study, since we did not carry out the analysis of the different fractions of PL.

The inclusion of flaxseeds and PSO resulted in a decrease of arachidonic acid in the PL fraction. Belury and Kempa-Stecko [38], when analysing the liver fatty acid composition of mice fed with dietary CLA, reported that because CLA compete with linoleic acid for Δ6-desaturase, diet-induced changes in the composition of phospholipid fatty acids were related to the partial replacement of arachidonic acid by other fatty acids such as n-3 PUFA. In our study, it appears that membrane-bound arachidonic acid has been replaced primarily by long-chain n-3 PUFA, as well as by PunA, RmA and c6,c9,t11-CLnA. The different biological effects of PunA, RmA and their metabolites may be partly explained by their incorporation into membrane structures where they can affect cellular characteristics and physiological processes, including membrane fluidity and transmembrane receptor function.

## 5. Conclusions

This study was designed to evaluate the impact of different dietary fatty acid sources (olive oil vs. flaxseeds + PSO) on egg yolk deposition and fatty acid composition of four important tissues (liver, heart, muscle and adipose tissue) of the hen. Supplementation with flaxseeds and PSO altered the fatty acid composition of egg yolks, as well as tissues in hens. Dietary PunA was converted into RmA, and both fatty acids were incorporated into egg yolks and hens’ tissues. Enrichment of the eggs with ALA, DHA, PunA and RmA was persistent, and a steady state of accumulation was achieved for most of the fatty acids after one month of feeding. In addition, fatty acids derived from RmA were also detected in eggs and hens’ tissues. Taken together, these results indicate that combining flaxseeds with PSO is an effective way to produce eggs enriched with ALA, DHA, PunA and RmA and provide animal products with added nutritional value for human consumption.

## Figures and Tables

**Table 1:** Main fatty acid composition of fat sources and experimental diets.

Fatty Acids	Fat Sources			Dietary Treatments	
	Olive Oil	Flaxseed Oil	Pomegranate Seed Oil	F-OL	FS-PSO
	% wt. of total fatty acids identified			mg/g of feed	
Palmitic acid (C16:0)	9.68	5.27	2.61	19.36	9.08
Stearic acid (C18:0)	3.18	4.20	1.74	4.74	2.96
Oleic acid (C18:1c9)	78.46	16.66	4.39	91.06	14.74
Cis-vaccenic acid (C18:1c11)	1.61	0.67	0.40	2.16	0.67
Linoleic acid (C18:2c9,c12)	4.82	15.22	5.67	23.63	21.16
Alpha-linolenic acid (C18:3c9,c12,c15)	0.71	56.80	0.73	2.09	16.57
Punicic acid (C18:3c9,t11,c13)	ND	ND	73.21	ND	39.53
Alpha-eleostearic acid (C18:3c9,t11,t13) *	ND	ND	0.64	ND	0.35
Alpha-calendic acid (C18:3t8,t10,c12) *	ND	ND	0.82	ND	0.44
Catalpic acid (C18:3t9,t11,c13) *	ND	ND	4.85	ND	2.62
Beta-eleostearic acid (C18:3t9,t11,t13) *	ND	ND	1.99	ND	1.08
Beta-calendic acid (C18:3t8,t10,t12) *	ND	ND	0.56	ND	0.30
Σ SFA	13.56	9.99	5.70	25.74	13.53
Σ MUFA	80.76	17.44	5.51	94.26	15.52
Σ n-6 PUFA	4.82	15.27	5.70	23.63	21.26
Σ n-3 PUFA	0.84	57.26	1.02	2.25	16.69
Σ CLnA	ND	ND	82.07	ND	44.36

* Calculated as punicic acid equivalents. F-OL: hens’ feed supplemented with 10 wt % olive oil; FS-PSO: hens’ feed supplemented with 7.8 wt % flaxseeds and 7 wt % pomegranate seed oil; ND: not detected; Σ SFA: total saturated fatty acids; Σ MUFA: total monounsaturated fatty acids; Σ n-6 PUFA: total omega-6 polyunsaturated fatty acids; Σ n-3 PUFA: total omega-3 polyunsaturated fatty acids; Σ CLnA: total conjugated linolenic acids.

**Table 2:** Effect of dietary treatments on hens’ performance, egg weight and egg yolk weight.

		Diet Effect		Diet × Period Effect						*p*-Value		
	Dietary Treatments		SE	Period 1	Period 2	Period 3	Period 4	Period 5	Period 6	Diet	Period	Diet × Period
Laying rate (%)	F-OL	85.75	1.61	76.97	86.81	89.65	89.85	85.69	85.51	0.75	0.12	0.93
	FS-PSO	85.01		79.19	82.89	92.17	85.37	85.44	84.98			
Egg weight (g)	F-OL	62.86	0.47	63.41	65.54	60.92	60.97	62.63	63.64	0.06	0.06	0.11
	FS-PSO	61.60		60.11	61.04	62.34	60.14	62.44	63.53			
Yolk weight (g)	F-OL	16.57	0.13	16.71	17.28	16.06	16.07	16.51	16.78	0.07	0.05	0.12
	FS-PSO	16.24		15.84	16.09	16.43	15.85	16.46	16.75			

Each period is 28 days and represents the average value of 4 weeks (six eggs per treatment were analyzed separately each week). Data are presented as least square mean (LSM) ± standard error (SE) between treatments (diet effect) or as LSM between periods (diet × period effect). F-OL: hens’ feed supplemented with 10 wt % olive oil. FS-PSO: hens’ feed supplemented with 7.8 wt % flaxseeds and 7 wt % pomegranate seed oil.

**Table 3:** Effect of supplementing the hen’s diet with olive oil or flaxseeds and pomegranate seed oil on the fatty acid composition of the yolks.

		Diet Effect		Diet × Period Effect						*p*-Value		
Fatty Acids (In mg/g of Yolk)	Dietary Treatments		SE	Period 1	Period 2	Period 3	Period 4	Period 5	Period 6	Diet	Period	Diet × Period
Palmitic acid (C16:0)	F-OL	59.15 ^a^	0.77	75.85 ^a^	60.04 ^b,c,d^	53.50 ^d^	56.98 ^d^	56.57 ^d^	51.93 ^d^	0.0031	<0.0001	0.0858
	FS-PSO	62.34 ^b^		74.49 ^a^	65.56 ^b^	59.44 ^b,c,d^	64.34 ^b,c^	57.34 ^c,d^	52.88 ^d^			
Hypogeic acid (C16:1c7)	F-OL	4.98 ^a^	0.09	5.14 ^a,b^	4.57 ^b^	4.72 ^a,b^	5.33 ^a,b^	5.55 ^a^	4.58 ^b^	<0.0001	0.0045	0.0186
	FS-PSO	2.38 ^b^		3.04 ^c^	2.10 ^c^	2.32 ^c^	2.27 ^c^	2.24 ^c^	2.32 ^c^			
Palmitoleic acid (C16:1c9)	F-OL	3.72 ^a^	0.11	6.90 ^a^	3.54 ^b^	2.94 ^b,c^	3.09 ^b,c^	3.24 ^b,c^	2.57 ^b,c^	0.0667	<0.0001	0.1002
	FS-PSO	3.43 ^a^		5.96 ^a,b^	3.73 ^b^	2.82 ^b,c^	3.40 ^b^	2.57 ^b,c^	2.08 ^c^			
Cis-7, trans-9-hexadecadienoic acid (C16:2c7,t9)	F-OL	0.07 ^a^	0.07	0.12 ^c^	0.06 ^c^	0.06 ^c^	0.07 ^c^	0.06 ^c^	Traces ^c^	<0.0001	0.0173	0.0059
	FS-PSO	3.75 ^b^		2.82 ^b^	3.64 ^a,b^	3.88 ^a^	3.77 ^a^	4.08 ^a^	4.31 ^a^			
Stearic acid (C18:0)	F-OL	20.27 ^a^	0.31	24.32 ^a^	19.89 ^b,c^	18.84 ^c^	20.36 ^b,c^	19.62 ^c^	18.62 ^c^	<0.0001	<0.0001	0.2497
	FS-PSO	23.49 ^b^		25.26 ^a^	24.63 ^a^	22.49 ^a,b,c^	24.22 ^a^	23.01 ^a,b^	21.32 ^a,b,c^			
Oleic acid (C18:1c9)	F-OL	153.40 ^a^	1.70	154.86 ^a^	160.25 ^a^	144.25 ^a^	158.13 ^a^	156.14 ^a^	146.78 ^a^	<0.0001	<0.0001	<0.0001
	FS-PSO	72.59 ^b^		103.87 ^b^	70.04 ^c^	67.19 ^c^	71.52 ^c^	63.30 ^c^	59.68 ^c^			
Cis-vaccenic acid (C18:1c11)	F-OL	4.30 ^a^	0.05	5.78 ^a^	4.25 ^b^	4.00 ^b,c^	4.16 ^b,c^	4.03 ^b,c^	3.56 ^c^	<0.0001	<0.0001	0.3349
	FS-PSO	2.45 ^b^		4.23 ^b,c^	2.34 ^d^	2.28 ^d^	2.29 ^d^	1.86 ^d^	1.68 ^d^			
Linoleic acid (C18:2c9,c12)	F-OL	33.11 ^a^	0.63	45.48 ^a,b^	30.17 ^d,e^	29.70 ^d,e^	32.67 ^c,d,e^	31.75 ^d,e^	28.86 ^e^	<0.0001	<0.0001	0.4329
	FS-PSO	39.46 ^b^		49.45 ^a^	36.65 ^c,d^	39.63 ^b,c^	37.98 ^c^	36.15 ^c,d,e^	36.89 ^c,d,e^			
Rumenic acid (C18:2c9,t11)	F-OL	0.13 ^a^	0.47	0.28 ^c^	0.16 ^c^	0.08 ^c^	0.08 ^c^	0.15 ^c^	0.06 ^c^	<0.0001	0.0026	0.0016
	FS-PSO	35.93 ^b^		28.64 ^b^	35.58 ^a^	35.25 ^a,b^	37.70 ^a^	38.23 ^a^	40.19 ^a^			
Trans-9, trans-11-octadecadienoic acid (C18:2t9,t11) *	F-OL	ND ^a^	0.02	ND ^a^	ND ^a^	ND ^a^	ND ^a^	ND ^a^	ND ^a^	<0.0001	0.6133	0.4942
	FS-PSO	0.96 ^b^		1.05 ^b^	1.01 ^b^	0.92 ^b^	0.92 ^b^	0.92 ^b^	0.92 ^b^			
Cis-6, cis-9, trans-11-octadecatrienoic acid (C18:3c6,c9,t11)	F-OL	0.05 ^a^	0.02	0.07 ^b^	0.06 ^b^	ND ^b^	0.07 ^b^	0.06 ^b^	0.05 ^b^	<0.0001	0.7764	0.7149
	FS-PSO	0.88 ^b^		0.94 ^a^	0.89 ^a^	0.94 ^a^	0.82 ^a^	0.84 ^a^	0.84 ^a^			
Alpha-linolenic acid (C18:3c9,c12,c15)	F-OL	0.91 ^a^	0.19	1.50 ^c^	0.86 ^c^	0.74 ^c^	0.84 ^c^	0.85 ^c^	0.67 ^c^	<0.0001	0.4431	0.0327
	FS-PSO	6.15 ^b^		4.76 ^b^	6.85 ^a,b^	5.61 ^a,b^	6.03 ^a,b^	6.35 ^a,b^	7.29 ^a^			
Punicic acid (C18:3c9,t11,c13)	F-OL	ND ^a^	0.40	ND ^c^	ND ^c^	ND ^c^	ND ^c^	ND ^c^	ND ^c^	<0.0001	0.0007	0.0007
	FS-PSO	20.00 ^b^		21.47 ^a,b^	19.14 ^b^	16.93 ^b^	18.05 ^b^	24.16 ^a^	20.23 ^a,b^			
Alpha-eleostearic acid (C18:3c9,t11,t13) **	F-OL	ND ^a^	0.004	ND ^d^	ND ^d^	ND ^d^	ND ^d^	ND ^d^	ND ^d^	<0.0001	0.0023	0.0023
	FS-PSO	0.25 ^b^		0.30 ^a^	0.26 ^a,b^	0.26 ^a,b^	0.21 ^c^	0.25 ^a,b,c^	0.23 ^b,c^			
Alpha-calendic acid (C18:3t8,t10,c12) **	F-OL	ND ^a^	0.004	ND ^d^	ND ^d^	ND ^d^	ND ^d^	ND ^d^	ND ^d^	<0.0001	<0.0001	<0.0001
	FS-PSO	0.20 ^b^		0.25 ^a,b^	0.26 ^a^	0.20 ^b^	0.23 ^a,b^	0.14 ^c^	0.12 ^c^			
Catalpic acid (C18:3t9,t11,c13) **	F-OL	ND ^a^	0.02	ND ^c^	ND ^c^	ND ^c^	ND ^c^	ND ^c^	ND ^c^	<0.0001	0.1392	0.1426
	FS-PSO	1.06 ^b^		1.18 ^a,b^	1.05 ^a,b^	1.16 ^a^	0.91 ^b^	1.07 ^a,b^	1.02 ^a,b^			
Beta-eleostearic acid (C18:3t9,t11,t13) **	F-OL	ND ^a^	0.01	ND ^c^	ND ^c^	ND ^c^	ND ^c^	ND ^c^	ND ^c^	<0.0001	0.0597	0.0600
	FS-PSO	0.69 ^b^		0.75 ^a,b^	0.68 ^a,b^	0.69 ^a,b^	0.58 ^b^	0.74 ^a^	0.70 ^a,b^			
Beta-calendic acid (C18:3t8,t10,t12) **	F-OL	ND ^a^	0.01	ND ^c^	ND ^c^	ND ^c^	ND ^c^	ND ^c^	ND ^c^	<0.0001	0.1362	0.1362
	FS-PSO	0.33 ^b^		0.38 ^a^	0.34 ^a,b^	0.34 ^a,b^	0.29 ^b^	0.34 ^a,b^	0.32 ^a,b^			
Arachidonic acid (C20:4c5,c8,c11,c14)	F-OL	5.66 ^a^	0.08	5.91 ^a,b^	5.42 ^a,b^	5.34 ^a,b,c^	5.86 ^a,b^	6.01 ^a^	5.40 ^a,b,c^	<0.0001	<0.0001	<0.0001
	FS-PSO	3.95 ^b^		5.05 ^b,c^	3.78 ^d,e^	4.45 ^c,d^	3.82 ^d,e^	3.61 ^d,e^	3.00^e^			
Eicosapentaenoic acid (C20:5c5,c8,c11,c14,c17)	F-OL	ND ^a^	0.005	ND ^c^	ND ^c^	ND ^c^	ND ^c^	ND ^c^	ND ^c^	<0.0001	0.1236	0.1289
	FS-PSO	0.16 ^b^		0.19 ^a^	0.18 ^a,b^	0.12 ^b^	0.16 ^a,b^	0.16 ^a,b^	0.15 ^a,b^			
n-6 Docosapentaenoic acid (C22:5c4,c7,c10,c13,c16)	F-OL	1.36 ^a^	0.03	1.47 ^a,b^	1.18 ^b^	1.52 ^a,b^	1.51 ^a^	1.33 ^a,b^	1.16 ^b,c^	<0.0001	<0.0001	0.0012
	FS-PSO	0.35 ^b^		0.80 ^c^	0.31 ^d^	0.34 ^d^	0.29 ^d^	0.20 ^d^	0.16 ^d^			
n-3 Docosapentaenoic acid (C22:5c7,c10,c13,c16,c19)	F-OL	0.48 ^a^	0.40	0.44 ^d^	0.41 ^d^	0.49 ^c,d^	0.56 ^c,d^	0.53 ^c,d^	0.41 ^d^	<0.0001	<0.0001	0.0002
	FS-PSO	1.39 ^b^		0.92 ^b,c^	1.72 ^a^	1.25 ^a,b^	1.44 ^a^	1.66 ^a^	1.35 ^a,b^			
Docosahexaenoic acid (C22:6c4,c7c10,c13,c16,c19)	F-OL	2.22 ^a^	0.10	2.57 ^c^	2.22 ^c^	2.27 ^c^	2.28 ^c^	2.08 ^c^	1.95 ^c^	<0.0001	0.0898	0.0020
	FS-PSO	4.90 ^b^		3.82 ^b^	5.06 ^a^	5.54 ^a^	5.14 ^a^	4.80 ^a,b^	5.03 ^a,b^			
Σ SFA	F-OL	81.79 ^a^	1.02	103.21 ^a^	82.50 ^d,e^	74.47^e^	79.72^e^	78.32^e^	72.51^e^	<0.0001	<0.0001	0.0934
	FS-PSO	88.87 ^b^		102.89 ^a,b^	93.48 ^a,b,c^	84.98 ^c,d,e^	91.75 ^b,c,d^	83.23 ^c,d,e^	76.86^e^			
Σ MUFA	F-OL	166.99 ^a^	1.87	171.46 ^a^	173.58 ^a^	156.68 ^a^	171.65 ^a^	170.07 ^a^	158.49 ^a^	<0.0001	<0.0001	0.0001
	FS-PSO	80.95 ^b^		116.79 ^b^	79.01 ^c^	69.58 ^c^	80.40 ^c^	70.81 ^c^	69.12 ^c^			
Σ n-6 PUFA	F-OL	42.03 ^a^	0.71	56.63 ^a^	38.18 ^b,c^	38.18 ^b,c^	41.78 ^b,c^	40.67 ^b,c^	36.76 ^c^	0.0015	<0.0001	0.2487
	FS-PSO	45.28 ^b^		56.43 ^a^	42.37 ^b,c^	46.23 ^b^	43.80 ^b,c^	41.45 ^b,c^	41.38 ^b,c^			
Σ n-3 PUFA	F-OL	3.67 ^a^	0.30	4.53 ^a^	3.55 ^a^	3.57 ^a^	3.74 ^a^	3.54 ^a^	3.08 ^a^	<0.0001	0.6020	0.5341
	FS-PSO	14.14 ^b^		14.55 ^b^	14.89 ^b^	12.73 ^b^	13.87 ^b^	14.00 ^b^	14.81 ^b^			
Σ CLA	F-OL	0.20 ^a^	0.46	0.38 ^c^	0.22 ^c^	0.13 ^c^	0.15 ^c^	0.21 ^c^	0.13 ^c^	<0.0001	0.0016	0.0007
	FS-PSO	37.04 ^b^		29.87 ^b^	36.79 ^a^	36.20 ^a^	38.81 ^a^	40.40 ^a^	41.29 ^a^			
Σ CLnA	F-OL	0.05 ^a^	0.39	0.07 ^c^	0.06 ^c^	ND ^c^	0.07 ^c^	0.06 ^c^	0.05 ^c^	<0.0001	0.0001	0.0002
	FS-PSO	23.34 ^b^		25.28 ^a,b^	22.62 ^b^	20.30 ^b^	20.85 ^b^	27.53 ^a^	23.47 ^a,b^			

Each period is 28 days and represents the average value of 4 weeks (six eggs per treatment were analyzed separately each week). Data are presented as least square mean (LSM) ± standard error (SE) between treatments (diet effect) or as LSM between periods (diet × period effect). Values with no common superscript letters (a, b, c, d, e) between treatments (diet effect) or between periods (diet × period effect) are significantly different at *p* ˂ 0.05, using 2-way ANOVA followed by Tukey’s test. F-OL: hens’ feed supplemented with 10 wt % olive oil. FS-PSO: hens’ feed supplemented with 7.8 wt % flaxseeds and 7 wt % pomegranate seed oil. * Calculated as rumenic acid equivalents. ** Calculated as punicic acid equivalents. “Traces” means detectable but too low to be quantified. ND: not detected; Σ SFA: total saturated fatty acids; Σ MUFA: total monounsaturated fatty acids; Σ n-6 PUFA: total omega-6 polyunsaturated fatty acids; Σ n-3 PUFA: total omega-3 polyunsaturated fatty acids; Σ CLA: total conjugated linoleic acids; Σ CLnA: total conjugated linolenic acids.

**Table 4:** Effect of dietary fats on fatty acid composition of triglycerides and phospholipids of yolk.

	Triglycerides		Phospholipids	
% wt. of total fatty acids identified	F-OL	FS-PS	F-OL	FS-PS
Palmitic acid (C16:0)	17.91 ± 1.93 ^a^	19.92 ± 1.32 ^b^	25.07 ± 0.35 ^a^	23.40 ± 0.49 ^b^
Hypogeic acid (C16:1c7)	2.38 ± 0.12 ^a^	0.96 ± 0.09 ^b^	0.56 ± 0.03 ^a^	0.17 ± 0.01 ^b^
Palmitoleic acid (C16:1c9)	1.12 ± 0.08 ^a^	1.09 ± 0.07 ^a^	0.41 ± 0.02 ^a^	0.27 ± 0.01 ^b^
Cis-7, trans-9-hexadecadienoic acid (C16:2cis7,t9)	0.01 ± 0.004 ^a^	1.87 ± 0.27 ^b^	0.02 ± 0.002 ^a^	0.20 ± 0.01 ^a^
Stearic acid (C18:0)	4.22 ± 0.20 ^a^	5.60 ± 0.21 ^b^	17.83 ± 0.50 ^a^	19.39 ± 0.45 ^b^
Oleic acid (C18:1c9)	30.45 ± 9.19 ^a^	17.90 ± 3.84 ^b^	28.34 ± 0.55 ^a^	12.58 ± 0.36 ^b^
Cis-vaccenic acid (C18:1c11)	30.08 ± 8.64 ^a^	8.85 ± 3.47 ^b^	1.12 ± 0.04 ^a^	0.38 ± 0.01 ^b^
Linoleic acid (C18:2c9,c12)	11.02 ± 0.34 ^a^	13.62 ± 0.87 ^a^	12.39 ± 0.31 ^a^	12.04 ± 0.26 ^a^
Rumenic acid (C18:2c9,t11)	0.06 ± 0.01 ^a^	14.62 ± 0.41 ^b^	0.04 ± 0.01 ^a^	9.64 ± 0.32 ^b^
Trans-9, trans-11-octadecadienoic acid (C18:2t9,t11) *	ND ^a^	0.01 ± 0.005 ^b^	ND ^a^	ND ^a^
Cis-6, cis-9, trans-11-octadecatrienoic acid (C18:3c6,c9,t11)	0.01 ± 0.004 ^a^	0.24 ± 0.010 ^b^	0.01 ± 0.002 ^a^	0.56 ± 0.03 ^b^
Alpha-linolenic acid (C18:3c9,c12,c15)	0.30 ± 0.01 ^a^	2.89 ± 0.24 ^b^	0.11 ± 0.004 ^a^	0.60 ± 0.04 ^b^
Punicic acid (C18:3c9,t11,c13)	ND ^a^	7.90 ± 1.15 ^b^	ND ^a^	4.98 ± 0.3 ^b^
Alpha-eleostearic acid (C18:3c9,t11,t13) **	ND ^a^	0.08 ± 0.004 ^b^	ND ^a^	0.05 ± 0.002 ^b^
Alpha-calendic acid (C18:3t8,t10,c12) **	ND ^a^	0.06 ± 0.01 ^b^	ND ^a^	0.05 ± 0.004 ^b^
Catalpic acid (C18:3t9,t11,c13) **	ND ^a^	0.35 ± 0.07 ^b^	ND ^a^	0.28 ± 0.01 ^b^
Beta-eleostearic acid (C18:3t9,t11,t13) **	ND ^a^	0.24 ± 0.02 ^b^	ND ^a^	0.19 ± 0.01 ^b^
Beta-calendic acid (C18:3t8,t10,t12) **	ND ^a^	0.11 ± 0.01 ^b^	ND ^a^	0.07 ± 0.004 ^b^
Arachidonic acid (C20:4c5,c8,c11,c14)	0.72 ± 0.10 ^a^	0.41 ± 0.06 ^b^	6.48 ± 0.28 ^a^	4.51 ± 0.14 ^b^
Eicosapentaenoic acid (C20:5c5,c8,c11,c14,c17)	ND ^a^	0.05 ± 0.01 ^b^	ND ^a^	0.16 ± 0.01 ^b^
n-6 Docosapentaenoic acid (C22:5c4,c7,c10,c13,c16)	0.13 ± 0.03 ^a^	0.03 ± 0.01 ^b^	1.40 ± 0.19 ^a^	0.26 ± 0.03 ^b^
n-3 Docosapentaenoic acid (C22:5c7,c10,c13,c16,c19)	0.12 ± 0.008 ^a^	0.39 ± 0.03 ^b^	0.43 ± 0.01 ^a^	1.41 ± 0.10 ^b^
Docosahexaenoic acid (C22:6c4,c7c10,c13,c16,c19)	0.20 ± 0.03 ^a^	0.48 ± 0.07 ^b^	2.83 ± 0.13 ^a^	6.55 ± 0.38 ^b^
Σ SFA	22.48 ± 0.63 ^a^	26.20 ± 0.49 ^b^	44.00 ± 0.86 ^a^	43.35 ± 0.92 ^a^
Σ MUFA	64.44 ± 0.81 ^a^	29.01 ± 0.73 ^b^	30.90 ± 0.57 ^a^	13.60 ± 0.32 ^b^
Σ n-6 PUFA	12.25 ± 0.43 ^a^	14.52 ± 0.27 ^b^	21.44 ± 0.38 ^a^	17.71 ± 0.35 ^b^
Σ n-3 PUFA	0.64 ± 0.03 ^a^	3.91 ± 0.23 ^b^	3.41 ± 0.12 ^a^	8.83 ± 0.43 ^b^
Σ CLA	0.07 ± 0.01 ^a^	14.67 ± 0.43 ^b^	0.05 ± 0.01 ^a^	9.66 ± 0.33 ^b^
Σ CLnA	0.01 ± 0.004 ^a^	8.98 ± 0.33 ^b^	0.01 ± 0.002 ^a^	6.18 ± 0.28 ^b^

Data are means ± standard errors of the mean (*n* = 24). Values with no common superscript letters (a, b) are significantly different at *p* ˂ 0.05, using two-sample *t*-test. F-OL: hens’ feed supplemented with 10 wt % olive oil. FS-PSO: hens’ feed supplemented with 7.8 wt % flaxseeds and 7 wt % pomegranate seed oil. * Calculated as rumenic acid equivalents. ** Calculated as punicic acid equivalents. ND: not detected; Σ SFA: total saturated fatty acids; Σ MUFA: total monounsaturated fatty acids; Σ n-6 PUFA: total omega-6 polyunsaturated fatty acids; Σ n-3 PUFA: total omega-3 polyunsaturated fatty acids; Σ CLA: total conjugated linoleic acids; Σ CLnA: total conjugated linolenic acids.

**Table 5:** Fatty acid composition of triglycerides and phospholipids in the liver, heart, adipose tissue and muscle of hens fed with the experimental diets.

		Liver		Heart		Muscle		Adipose Tissue	
% wt. of total fatty acids identified	Lipid fractions	F-OL	FS-PSO	F-OL	FS-PSO	F-OL	FS-PSO	F-OL	FS-PSO
Palmitic acid (C16:0)	TG	16.55 ± 0.77 ^a^	18.15 ± 1.02 ^a^	12.23 ± 0.34 ^a^	14.25 ± 0.34 ^b^	13.99 ± 0.41 ^a^	15.59 ± 0.49 ^a^	11.04 ± 0.29 ^a^	13.62 ± 0.29 ^b^
	PL	22.14 ± 0.15 ^a^	20.90 ± 0.51 ^b^	20.48 ± 0.36 ^a^	19.49 ± 0.46 ^a^	17.88 ± 0.43 ^a^	18.09 ± 0.80 ^a^	ND	ND
Hypogeic acid (C16:1c7)	TG	1.53 ± 0.10 ^a^	0.70 ± 0.08 ^b^	0.66 ± 0.04 ^a^	0.47 ± 0.05 ^b^	0.94 ± 0.06 ^a^	0.65 ± 0.04 ^b^	0.52 ± 0.03 ^a^	0.39 ± 0.03 ^b^
	PL	0.41 ± 0.03 ^a^	0.16 ± 0.02 ^b^	0.14 ± 0.005 ^a^	0.07 ± 0.01 ^b^	0.14 ± 0.01 ^a^	0.05 ± 0.01 ^b^	ND	ND
Palmitoleic acid (C16:1c9)	TG	0.91 ± 0.16 ^a^	0.83 ± 0.04 ^a^	0.85 ± 0.10 ^a^	0.98 ± 0.11 ^a^	0.89 ± 0.11 ^a^	1.34 ± 0.22 ^a^	0.64 ± 0.06 ^a^	0.80 ± 0.06 ^a^
	PL	0.37 ± 0.06 ^a^	0.28 ± 0.02 ^a^	0.10 ± 0.01 ^a^	0.08 ± 0.01 ^a^	0.12 ± 0.004 ^a^	0.13 ± 0.01 ^a^	ND	ND
Stearic acid (C18:0)	TG	5.19 ± 0.30 ^a^	7.22 ± 0.50 ^b^	3.21 ± 0.11 ^a^	4.20 ± 0.25 ^b^	4.27 ± 0.13 ^a^	5.92 ± 0.92 ^a^	3.30 ± 0.13 ^a^	4.49 ± 0.42 ^b^
	PL	18.54 ± 0.55 ^a^	17.13 ± 0.88 ^a^	20.76 ± 0.61 ^a^	20.74 ± 0.75 ^a^	18.65 ± 0.49 ^a^	16.89 ± 1.24 ^a^	ND	ND
Oleic acid (C18:1c9)	TG	60.22 ± 0.65 ^a^	27.91 ± 1.88 ^b^	48.28 ± 0.30 ^a^	33.23 ± 1.18 ^b^	58.49 ± 0.45 ^a^	35.14 ± 1.66 ^b^	63.42 ± 0.24 ^a^	30.88 ± 2.01 ^b^
	PL	26.16 ± 0.68 ^a^	12.12 ± 0.85 ^b^	13.65 ± 0.49 ^a^	5.19 ± 0.57 ^b^	21.10 ± 0.35 ^a^	5.84 ± 0.58 ^b^	ND	ND
Cis-vaccenic acid (C18:1c11)	TG	1.37 ± 0.07 ^a^	1.03 ± 0.08 ^b^	14.76 ± 0.09 ^a^	1.23 ± 0.05 ^b^	1.84 ± 0.02 ^a^	1.52 ± 0.11 ^b^	2.47 ± 0.11 ^a^	1.69 ± 0.12 ^b^
	PL	1.04 ± 0.03 ^a^	0.44 ± 0.04 ^b^	2.00 ± 0.16 ^a^	0.69 ± 0.10 ^b^	2.07 ± 0.05 ^a^	0.82 ± 0.08 ^b^	ND	ND
Linoleic acid (C18:2c9,c12)	TG	11.59 ± 1.23 ^a^	17.60 ± 0.37 ^b^	17.77 ± 0.55 ^a^	23.85 ± 0.67 ^b^	17.05 ± 0.68 ^a^	20.05 ± 0.78 ^b^	16.40 ± 0.33 ^a^	23.63 ± 0.60 ^b^
	PL	11.66 ± 0.36 ^a^	11.51 ± 0.45 ^a^	10.61 ± 0.54 ^a^	13.71 ± 0.53 ^b^	16.02± 0.75 ^a^	24.15 ± 0.81 ^b^	ND	ND
Rumenic acid (C18:2c9,t11)	TG	0.02 ± 0.004 ^a^	9.88 ± 1.09 ^b^	0.01 ± 0.001 ^a^	9.04 ± 0.85 ^b^	0.02 ± 0.003 ^a^	8.68 ± 1.05 ^b^	0.01 ± 0.005 ^a^	10.07 ± 1.33 ^b^
	PL	0.02 ± 0.001 ^a^	8.34 ± 1.10 ^b^	Traces ^a^	5.73 ± 0.47 ^b^	0.02 ± 0.01 ^a^	5.26 ± 0.48 ^b^	ND	ND
Cis-6, cis-9, trans-11-octadecatrienoic acid (C18:3c6,c9,t11)	TG	ND ^a^	1.72 ± 0.26 ^b^	ND ^a^	1.38 ± 0.14 ^b^	ND ^a^	1.26 ± 0.13 ^b^	ND ^a^	1.94 ± 0.21 ^b^
	PL	ND ^a^	3.80 ± 0.92b	ND ^a^	2.14 ± 0.44 ^b^	ND ^a^	2.42 ± 0.23 ^b^	ND	ND
Alpha-linolenic acid (C18:3c9,c12,c15)	TG	0.39 ± 0.07 ^a^	3.92 ± 1.08 ^b^	0.73 ± 0.04 ^a^	5.93 ± 1.27 ^b^	0.73 ± 0.05 ^a^	4.05 ± 0.71 ^b^	1.09 ± 0.04 ^a^	5.96 ± 1.32 ^b^
	PL	0.09 ± 0.02 ^a^	0.47 ± 0.09 ^b^	0.08 ± 0.002 ^a^	0.29 ± 0.07 ^b^	0.12 ± 0.005 ^a^	0.45 ± 0.11 ^b^	ND	ND
Punicic acid (C18:3c9,t11,c13)	TG	ND ^a^	4.69 ± 0.99 ^b^	ND ^a^	1.38 ± 0.43 ^b^	ND ^a^	1.38 ± 0.29 ^b^	ND ^a^	1.44 ± 0.37 ^b^
	PL	ND ^a^	3.18 ± 0.70b	ND ^a^	0.67 ± 0.10 ^b^	ND ^a^	0.91 ± 0.28 ^b^	ND	ND
Arachidonic acid (C20:4c5,c8,c11,c14)	TG	0.36 ± 0.07 ^a^	0.33 ± 0.05 ^a^	0.14 ± 0.01 ^a^	0.12 ± 0.02 ^a^	0.15 ± 0.03 ^a^	0.12 ± 0.03 ^a^	0.08 ± 0.01 ^a^	0.05 ± 0.01 ^a^
	PL	10.85 ± 0.30 ^a^	7.53 ± 0.82 ^b^	26.14 ± 1.48 ^a^	24.08 ± 1.08 ^a^	13.04 ± 0.89 ^a^	10.42 ± 1.41 ^a^	ND	ND
Eicosapentaenoic acid (C20:5c5,c8,c11,c14,c17)	TG	Traces ^a^	0.02 ± 0.01 ^b^	Traces ^a^	0.01 ± 0.001 ^a^	Traces ^a^	0.01 ± 0.01 ^b^	ND	ND
	PL	0.03 ± 0.002 ^a^	0.50 ± 0.16 ^b^	0.05 ± 0.01 ^a^	0.47 ± 0.16 ^b^	0.03 ± 0.004 ^a^	0.29 ± 0.08 ^b^	ND	ND
n-6 Docosapentaenoic acid (C22:5c4,c7,c10,c13,c16)	TG	0.06 ± 0.01 ^a^	ND ^b^	0.01 ± 0.001 ^a^	Traces ^b^	0.01 ± 0.004 ^a^	ND ^b^	ND	ND
	PL	1.98 ± 0.17 ^a^	0.20 ± 0.05 ^b^	0.91 ± 0.06 ^a^	0.09 ± 0.02 ^b^	1.60 ± 0.19 ^a^	0.16 ± 0.03 ^b^	ND	ND
n-3 Docosapentaenoic acid (C22:5c7,c10,c13,c16,c19)	TG	0.04 ± 0.01 ^a^	0.16 ± 0.02 ^b^	0.01 ± 0.002 ^a^	0.03 ± 0.005 ^a^	0.02 ± 0.005 ^a^	0.04 ± 0.01 ^a^	ND ^a^	0.01 ± 0.01 ^b^
	PL	0.40 ± 0.05 ^a^	1.40 ± 0.15 ^b^	0.32 ± 0.04 ^a^	0.95 ± 0.09 ^b^	0.86 ± 0.09 ^a^	1.76 ± 0.16 ^b^	ND	ND
Docosahexaenoic acid (C22:6c4,c7c10,c13,c16,c19)	TG	0.11 ± 0.02 ^a^	0.30 ± 0.02 ^b^	0.02 ± 0.001 ^a^	0.04 ± 0.004 ^a^	0.04 ± 0.01 ^a^	0.08 ± 0.02 ^a^	ND ^a^	0.01 ± 0.01 ^b^
	PL	3.80 ± 0.46 ^a^	8.30 ± 0.84 ^b^	0.59 ± 0.04 ^a^	1.42 ± 0.11 ^b^	4.13 ± 0.17 ^a^	7.61 ± 0.32 ^b^	ND	ND
Σ SFA	TG	22.55 ± 0.84 ^a^	26.83 ± 1.43 ^b^	15.99 ± 0.42 ^a^	19.34 ± 0.56 ^b^	19.03 ± 0.34 ^a^	23.00 ± 1.61 ^b^	15.18 ± 0.34 ^a^	19.33 ± 0.74 ^b^
	PL	41.46 ± 0.48 ^a^	38.75 ± 1.03 ^b^	43.27 ± 0.97 ^a^	42.50 ± 0.77 ^a^	35.43 ± 3.55 ^a^	37.42 ± 0.61 ^a^	ND	ND
Σ MUFA	TG	64.50 ± 0.81 ^a^	30.98 ± 2.00 ^b^	65.08 ± 0.41 ^a^	36.17 ± 1.33 ^b^	62.44 ± 0.59 ^a^	39.07 ± 1.84 ^b^	67.10 ± 0.18 ^a^	34.16 ± 2.13 ^b^
	PL	28.30 ± 0.79 ^a^	13.19 ± 0.89 ^b^	16.14 ± 0.38 ^a^	6.18 ± 0.64 ^b^	30.08 ± 6.38 ^a^	7.08 ± 0.65 ^b^	ND	ND
Σ n-6 PUFA	TG	12.32 ± 1.35 ^a^	18.29 ± 0.35 ^b^	18.09 ± 0.55 ^a^	24.19 ± 0.68 ^b^	17.44 ± 0.73 ^a^	20.41 ± 0.79 ^b^	16.56 ± 0.33 ^a^	23.87 ± 0.62 ^b^
	PL	25.82 ± 0.71 ^a^	20.30 ± 1.20 ^b^	39.43 ± 1.08 ^a^	38.76 ± 0.86 ^a^	29.88 ± 2.23 ^a^	35.73 ± 0.89 ^b^	ND	ND
Σ n-3 PUFA	TG	0.54 ± 0.11 ^a^	4.52 ± 1.10 ^b^	0.76 ± 0.04 ^a^	6.05 ± 1.27 ^b^	0.79 ± 0.07 ^a^	4.20 ± 0.73 ^b^	1.09 ± 0.04 ^a^	5.98 ± 1.31 ^b^
	PL	4.35 ± 0.53 ^a^	10.97 ± 0.97 ^b^	1.06 ± 0.09 ^a^	3.25 ± 0.31 ^b^	4.45 ± 0.72 ^a^	10.23 ± 0.51 ^b^	ND	ND
Σ CLA	TG	0.02 ± 0.01 ^a^	9.99 ± 1.10 ^b^	0.02 ± 0.002 ^a^	9.12 ± 0.86 ^b^	0.04 ± 0.004 ^a^	8.79 ± 1.10 ^b^	0.01 ± 0.005 ^a^	10.23 ± 1.20 ^b^
	PL	0.02 ± 0.002 ^a^	8.40 ± 1.10 ^b^	Traces ^a^	5.85 ± 0.48 ^b^	0.03 ± 0.01 ^a^	5.48 ± 0.50 ^b^	ND	ND
Σ CLnA	TG	ND ^a^	8.25 ± 1.57 ^b^	ND ^a^	4.15 ± 0.30 ^b^	ND ^a^	3.59 ± 0.33 ^b^	ND ^a^	4.72 ± 0.46 ^b^
	PL	ND ^a^	7.84 ± 1.73 ^b^	ND ^a^	3.00 ± 0.52 ^b^	ND ^a^	3.75 ± 0.58 ^b^	ND	ND

Data are means ± standard errors of the mean (*n* = 5). Values with no common superscript letters (a, b) are significantly different at *p* ˂ 0.05, using two-sample t-test. F-OL: hens’ feed supplemented with 10 wt % olive oil. FS-PSO: hens’ feed supplemented with 7.8 wt % flaxseeds and 7 wt % pomegranate seed oil. “Traces” means detectable but too low to be quantified. TG: triglycerides; PL: phospholipids; ND: not detected; Σ SFA: total saturated fatty acids; Σ MUFA: total monounsaturated fatty acids; Σ n-6 PUFA: total omega-6 polyunsaturated fatty acids; Σ n-3 PUFA: total omega-3 polyunsaturated fatty acids; Σ CLA: total conjugated linoleic acids; Σ CLnA: total conjugated linolenic acids.

## Data Availability

The study did not report any data.
